# PostMod: sequence based prediction of kinase-specific phosphorylation sites with indirect relationship

**DOI:** 10.1186/1471-2105-11-S1-S10

**Published:** 2010-01-18

**Authors:** Inkyung Jung, Akihisa Matsuyama, Minoru Yoshida, Dongsup Kim

**Affiliations:** 1 Department of Bio and Brain Engineering, KAIST, Daejeon 305-701, S. Korea; 2 Chemical Genetics Laboratory, RIKEN, Wako, Saitama 351-0198, Japan; 3 KAIST Institute for BioCentury, KAIST, Daejeon 305-701, S. Korea

## Abstract

**Background:**

Post-translational modifications (PTMs) have a key role in regulating cell functions. Consequently, identification of PTM sites has a significant impact on understanding protein function and revealing cellular signal transductions. Especially, phosphorylation is a ubiquitous process with a large portion of proteins undergoing this modification. Experimental methods to identify phosphorylation sites are labor-intensive and of high-cost. With the exponentially growing protein sequence data, development of computational approaches to predict phosphorylation sites is highly desirable.

**Results:**

Here, we present a simple and effective method to recognize phosphorylation sites by combining sequence patterns and evolutionary information and by applying a novel noise-reducing algorithm. We suggested that considering long-range region surrounding a phosphorylation site is important for recognizing phosphorylation peptides. Also, from compared results to AutoMotif in 36 different kinase families, new method outperforms AutoMotif. The mean accuracy, precision, and recall of our method are 0.93, 0.67, and 0.40, respectively, whereas those of AutoMotif with a polynomial kernel are 0.91, 0.47, and 0.17, respectively. Also our method shows better or comparable performance in four main kinase groups, CDK, CK2, PKA, and PKC compared to six existing predictors.

**Conclusion:**

Our method is remarkable in that it is powerful and intuitive approach without need of a sophisticated training algorithm. Moreover, our method is generally applicable to other types of PTMs.

## Background

Post-translational modifications (PTMs) have important implication on the protein functions involved in signal transductions and many human diseases. Especially, phosphorylation is one of the most ubiquitous of these processes with a reported 30 ~50% of eukaryotic proteins undergoing this modification. For this reason, identifying phosphorylation sites is important for understanding functional role of proteins and cell signalling networks. In order to determine phosphorylation sites several experimental tools such as mass spectrometry have been used. Experimental efforts using those techniques have made it possible to construct several databases for phosphorylation sites, such as Phospho.ELM [1,2], PhosphoSite [3], and PhosPhAt [4]. However, those techniques are time-consuming and high cost approaches. Due to such practical limitation, an efficient computational algorithm to recognize phosphorylation sites is highly desirable.

Previously, several methods to predict phosphorylation sites have been developed by probing evolutionary information, using physicochemical properties, or searching motif patterns. The most successful algorithms are machine learning-based approaches. Using the artificial neural network (ANN) models, NetPhosYesat [5] predicts phosphorylation sites in yeast, and NePhosK [6] provides a sequence-based phosphorylation site prediction service. Examples of support vector machine (SVM)-based approaches are PredPhospho [7], AutoMotif [8,9], and kinasePhos2.0 [10] which trains SVM by using amino acid coupling patterns and solvent accessibility. Recently, probabilistic frameworks and new kernel methods were suggested. PPSP [11] used Bayesian decision theory to predict PK-specific phosphorylation sites, and SiteSeek [12] was implemented with a high search sensitivity by introducing a new adaptive locally-effective kernel method with hydrophobic information. In addition, conditional random field model was applied to predict kinase-specific phosphorylation [13].

Despite high performance of those machine learning or statistical approaches, development of simple, intuitive, and generally applicable algorithms has been pursued. A group-based approach, GPS, simply and intuitively recognizes phosphorylation sites by calculating peptide similarities with BLOSUM62 matrix and deciding which group is closest to the given peptide after clustering known peptide groups [14]. Our study aimed to develop a new algorithm by inventing a new scoring method, as well as by introducing an effective noise-reducing system, which can be applied to different types of modifications. We developed a new scoring scheme to measure the sequence similarity by combining pairwise sequence similarity scores and profile-profile alignment scores. Basic assumption was that physicochemical information, motif information, and evolutionary information could be retrieved by measuring sequence similarities. We also generalized the motif scoring method, which has been conventionally used for predicting phosphorylation sites, by performing profile-profile alignments with gaps. It turned out that such generalization significantly improved the prediction accuracy. Considering both features together, we developed a new peptide sequence similarity scoring method. We then applied a noise-reducing system exploiting indirect relationships among peptide sequences. When we tested our new method on 48 different kinase groups, the results indicated that the two innovative features of our present work, i.e., a new sequence similarity scoring method and the noise-reducing system, both contributed to the outstanding performance of the new method in recognizing phosphorylation sites correctly, showing better performance than AutoMotif which is one of the best-performing methods. Also, by testing unbiased data set we can achieve better or comparable performance compared to six existing predictors.

## Methods

### Datasets

We developed our new method using Phospho.ELM (released in December 2008) database [2]. The database contains experimentally validated phosphorylation sites for 254 different kinases. From the database we selected kinase groups which contained more than 20 known phosphorylation sites, resulting in 48 different kinase groups in our test set. To develop and evaluate the new method, positive (phosphorylation) and negative (non-phosphorylation) peptides were needed to make the 'reference set'. For a specific phosphorylation type, positive peptides were all peptides in Phospho.ELM database that had the same type of phosphorylation. Negative peptides were randomly selected from sequences which shared the same phosphorylation residue types with positive peptides. We selected negative peptides 10 times more than the number of positive peptides. The whole dataset can be downloaded from our web server.

### Peptide sequence similarity scoring scheme

Our scoring system was designed to give a high score when two peptides have high similarity, indicating that if a query peptide gets high scores with known phosphorylation peptides, the query peptide is predicted to be a peptide with the same type of phosphorylation. To calculate peptide similarities we combined two different sequence similarity measures, one using BLOSUM62 matrix [15] and the other using profile-profile alignment which contains evolutionary information. Both measures are widely used to calculate sequence similarities. We assumed that comparing sequence similarity with BLOSUM62 matrix could provide similarity measure for physicochemical properties of the two sequences and motif patterns indirectly. Similarity score using BLOSUM62 matrix, *S*_*BLOSUM*62_, between peptides *A *and *B *with fixed window size 7 was defined as

The *score(A*_*i*_, *B*_*i*_) is the substitution scores between two amino acids *A*_*i *_and *B*_*i *_in BLOSUM62 matrix. The window size 7 was determined by referencing a previously developed method, GPS [14]. If the candidate phosphorylation sites were near the N or C terminus, we represented the absent terminal sequences as *X*.

The second component of our new scoring scheme is the profile-profile alignment scores. The conventional way to measure sequence similarity for the purpose of predicting PTM sites is to use motif scoring methods where gapless alignments were typically assumed. We generalized the motif scoring method by performing profile-profile alignments allowing gaps. To calculate similarity scores based on profile-profile alignment we first generated the position specific scoring matrix (PSSM) and the position specific frequency matrix (PSFM) for a protein sequence which contained a given peptide by using PSI-BLAST [16]. We used blastpgp version 2.2.15 with default parameters except the options for the number of iterations (j = 5) and the cutoff E-value value (h = 0.001). Then we extracted PSSM and PSFM corresponding to the given peptide. Using both matrices we computed profile-profile alignment scores for the position *i *of a peptide *A *and the position *j *of a peptide *B *(*PPA*_*ij*_) as follows,

where, *f*_*ik *_and *f*_*jk *_are the frequencies of PSFM matrix at the position of *i *and *j*, and *S*_*ik *_and *S*_*jk *_represent the scores of PSSM matrix at the position of *i *and *j*. The detail procedure was reported in our previous work [17]. We aligned both peptides *A *and *B *and calculated similarity scores by using dynamic programming with gap penalty of 3.0 and gap extension penalty of 0.75. We referred to this profile-profile alignment scores as *S*_*profile*_. During profile-profile alignment we selected the window size as 41 after trying several different window sizes such as 7, 19, 41, and 101. In previous works, wide ranges of values from 7 to 19 have been used as a window size for calculating the sequence similarity. We increased the window size from 7 up to 101 to evaluate effect of considering long-range region surrounding phosphorylation sites. The performances for PKB-group with different window sizes were measured, and then 41 was selected as the optimal window size for calculating the profile-profile alignments. Once having calculated both types of similarity scores, *S*_*BLOSUM*62 _and *S*_*profile*_, we multiplied both scores to calculate the final similarity score (*S*_*combined*_) of the two peptides as follows,

The positive effect of combined-measure is described in Result and discussion section. We also tried a linear combination of *S*_*BLOSUM*62 _and *S*_*profile *_as the final similarity score and found that the multiplicative form of the two scores showed better performance.

### Noise reduction scheme utilizing indirect relationships

By using similarity scores we can rank all reference peptides for a given query peptide. If a scoring system is perfect, it would give higher scores to all true phosphorylation peptides than to any of non-phosphorylation peptides. However, our scoring system is obviously imperfect, partly because our current scoring system only considers sequence features. We may be able to improve the accuracy by adding new features, but in this work we focused on designing noise-reducing system by considering indirect relationships among reference peptides. Basic idea is that if highly ranked reference peptides tend to be known phosphorylation peptides, the query peptide is likely to be a phosphorylation site, otherwise a non-phosphorylation site.

**Figure 1:**
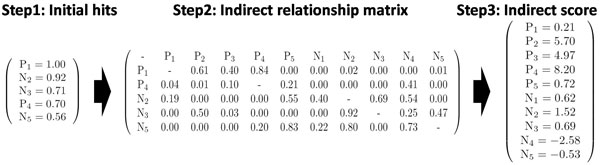
**Illustration of the noise-reducing system**. Illustration of the noise-reducing system. In step 1, we find the top 5 hits for a given query, where *P*_*j *_is a phosphorylation peptide and *N*_*j *_is a non-phosphorylation peptide. Next, *S*_*combined *_scores are calculated between the top 5 hits and all peptides in a reference set (10 peptides), where if a peptide *i *is not included in top 5 hits for a peptide *j *the score (*j*, *i*) is set to zero. In step 3, by summing each row of indirect relationship matrix we calculate indirect scores. During summation we assume that scores between positive (or negative) peptides are signal, while those between positive (or negative) and negative (or positive) are noise. Finally, we check the number of phosphorylation peptides among the top 4 hits by indirect scores. In this example P_2_, P_3_, P_4_, and N_2 _are recognized as the top 4 hits, and among them 3 peptides are phosphorylation peptides, and thereby we predict that the query peptide is a phosphorylation peptide.

Figure 1 illustrates the noise-reducing scoring system of this work. In step 1, for a given query (in this example, a phosphorylation peptide), we calculate *S*_*combined *_scores for all peptides in the reference set, and select the top α highly ranked peptides (in this example, α = 5, consisting of 2 phosphorylation peptides (*P*_*j*_) and 3 non-phosphorylation peptides (*N*_*j*_)). Next in step 2, indirect relationship matrix is constructed by calculating *S*_*combined *_scores between those top α hits and all peptides in the reference set (in this example, there are 5 phosphorylation and 5 non-phosphorylation peptides). When constructing the matrix, if a peptide *i *is not included in the top 5 hits for a peptide *j*, the score (*j*, *i*) of the matrix is set to zero. In step 3, indirect scores are calculated by using indirect relationship matrix. We assume that scores between positive (or negative) peptides are signal, while those between positive (or negative) and negative (or positive) are noise. According to our hypothesis, indirect scores can be calculated as follows,

where we give the weight of 10 to positive peptides since the number of negative peptides is 10 times that of positive peptides. For example, suppose that P_2 _recognized P_1 _and P_4 _as signal, and N_3 _as noise. Then, the final indirect score of P_2 _(5.70) is calculated by adding P_1 _and P_4 _with weight 10 (10*(0.61+0.01)) and subtracting N_3 _(0.50). Finally, from the indirect scores we consider the top β hits (in this example, β = 4) as query related peptides. Then, if the number of positive peptides are greater than γ (in this example, γ = 2), we predict the query peptide as a phosphorylation peptide. In this example P_2_, P_3_, P_4_, and N_2 _are the top 4 hits, and thereby we predict that the query peptide is a phosphorylation peptide.

There existed several parameter determination issues in constructing the noise-reducing system such as the number of highly ranked peptides α, the value of β and γ. To determine those parameters we searched the optimized parameter set using PKB-group. It is obvious that different kinase groups have the different optimized parameters but we applied the same parameters to all cases to avoid over-fitting. As a result, for the number of highly ranked peptides we selected the value of half number of positive peptides in the reference set. For the value of β and γ we used value of one-third and one-fourth of positive peptides, respectively.

### Performance assessment

We assessed the prediction performance with leave-one-out cross validation (LOOCV). We used all dataset except one as the reference set and tested our scoring system with left-out one peptide. The accuracy (ACC), precision (P), and recall (R) were calculated to measure the performance. The equations are as follows.

We define phosphorylation peptides as a positive class, and TP, TN, FP, and FN

denote true positives, true negatives, false positives, and false negatives, respectively. We benchmarked another prediction server, AutoMotif [8,9]. We directly compared the performance of our method to that of AutoMotif since our dataset and evaluation scheme were same as AutoMotif. The performance data of AutoMotif were extracted from website [18]. Furthermore, to test unbiased data set we used new data set constructed by Wan et al. [19]. We calculated ROC score defined as the areas under the ROC curves, the plot of true positives as a function of the number of false positives [20], and compared our result to PPSP, PredPhospho, GPS, KinasePhos 1.0, NetPhosK, and Scansite.

## Results and discussion

### Performance variation of various features for PKB-group

We evaluated the ability of different level of similarity measures to discriminate phosphorylation and non-phosphorylation peptides. As we described in Method section, we tested four different types of scores, *S*_*BLOSUM*62_, *S*_*profile*_, *S*_*combined*_, and our noise-reducing system. We selected PKB-group (protein kinase B) as a toy example to select parameters and to test performance variation of various features. The serine/threonine kinase PKB has been shown to play a crucial role in the control of diverse and important cellular functions such as cell survival and glycogen metabolism [21]. The result of performance comparison for PKB-group is shown in Table 1. In the aspect of precision and recall, *S*_*combined *_was significantly better than *S*_*BLOSUM*62 _and *S*_*profile*_. The precision and recall were increased more than 22% and 54%, respectively from those of *S*_*BLOSUM*62 _by combining *S*_*profile*_. This indicates that combining both similarity measures lead to significant positive effect. The positive effect is likely to be originated from removing ambiguous cases such as high score in *S*_*BLOSUM*62 _but low score in *S*_*profile*_, and vice versa. Furthermore, the noise-reducing system highly increased recall (24% increased) compared to *S*_*combined *_at the similar level of precisions (0.87 at Noise-reducing, 0.84 at *S*_*combined*_), indicating the noise-reducing system filtered out many false positives and rescued many true phosphorylation peptides which were falsely recognized as non-phosphorylation peptides by *S*_*combined*_.

**Table 1:** Performances of PKB-group kinases using different features

	*S*_*BLOSUM*62_	*S*_*profile*_	*S*_*combined*_	Noise-reducing
ACC	0.92	0.91	0.94	0.95
P	0.69	0.60	0.84	0.87
R	0.24	0.04	0.37	0.46

We also evaluated overall performance across 48 kinase groups. The result is summarized in Table 2, showing that in terms of overall performance our noise-reducing system is most effective for identifying phosphorylation sites. Especially, recall was increased about 15% from *S*_*combined *_at the 0.68 precision. Also significant performance enhancement was occurred in *S*_*combined *_compared to both *S*_*BLOSUM*62 _and *S*_*profile*_. The results remark that combining *S*_*profile *_with *S*_*BLOSUM*62 _is generally effective to increase discriminate ability for phosphorylation sites. Moreover, we expect that we can apply the same noise-reducing system to other types of post-translation modifications such as ubiquitination.

**Table 2:** Performances of 48 kinase groups using different features

	*S*_*BLOSUM*62_	*S*_*profile*_	*S*_*combined*_	Noise-reducing
ACC	0.92	0.91	0.93	0.93
P	0.59	0.43	0.68	0.68
R	0.24	0.31	0.34	0.39

To better understand how the noise-reducing system determines phosphorylation sites with higher accuracy, we consider one specific example, the phosphorylation peptide, [RGGSASRS]. The first and fourth hits (both are non-phosphorylation sites, false positives) of the given peptide are [DGTSLKV] and [VQDTYQI], respectively, with *S*_*combined*_. However, the first hit does not have any indirect relationships with other highly ranked peptides, thereby in the noise-reducing system its rank drops to the 38_th_. Also the fourth hit shows indirect relationships with highly ranked positive peptides (this is a false relationship), therefore the rank of third hit also drops to 40_th_. On the other hand, the ranks of several positive peptides are increased since several true indirect relationships exist among highly ranked positive peptides. This example shows exactly how the noise-reducing system works to detect phosphorylation sites.

### Importance of considering long-range region surrounding a phosphorylation site

Several mechanisms have been proposed to understand kinase specific binding properties. Protein kinase forms a protein complex with its substrate through recognizing phosphorylation binding domains or short sequence patterns of substrates. To recognize kinase-specific motifs, not only short sequence patterns but also local structure around a phosphorylation site may be important. We assume that this information can be measured by calculating similarities of long-range region surrounding phosphorylation sites with profile-profile alignment (PPA).

**Figure 2:**
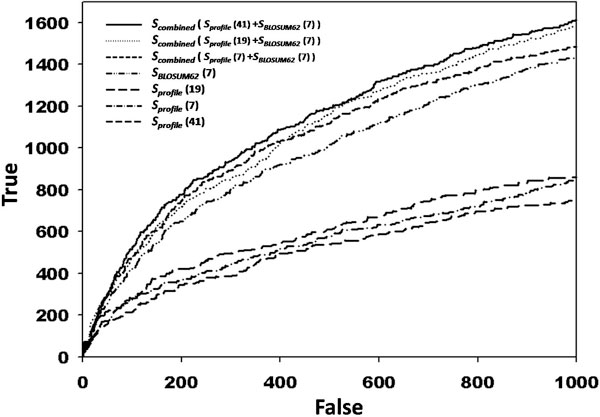
**ROC curves with various features**. The figure shows number of true matches (phosphorylation peptides) according to number of false matches (non-phosphorylation peptides) up to 1000 false matches among 48 kinase families. In figure, values in brackets represent number of windowed residues in peptides. From the figure we note that *S*_*combined *_with *S*_*profile *_(41 windowed residues) shows best performance. The fact remarks that considering long-range region is effective to identify phosphorylation peptides.

We evaluated our assumption by comparing performance with various features. We drew number of true matches (phosphorylation peptides) according to number of false matches (non-phosphorylation peptides) up to 1000 false matches among 48 kinase families. The results are shown in figure 2, and the performance varies according to window size of peptides. In figure, values in brackets represent number of windowed residues. Here, *S*_*BLOSUM*62 _represents a short sequence pattern comparison scheme with BLOSUM62 matrix for 7 windowed residues. When we extended window size as 41 the performance was degraded, therefore we fixed window size as 7 in *S*_*BLOSUM*62_. To evaluate effect of long-range similarities first, we searched proper window size of long-range region by using PPA based score (*S*_*profile*_) alone. Among three different window sizes, *S*_*profile *_with 19 windowed residues provided more precise similarities compared to 7 or 41 windowed residues. *S*_*profile *_(19 windowed residues), *S*_*profile *_(7 windowed residues), and *S*_*profile *_(41 windowed residues) recognized 610, 567, and 539 true matches up to 500 false matches. Next, we combined both *S*_*BLOSUM*62 _and *S*_*profile *_and measured performance variations. In this case, the best performance was occurred at the combination of *S*_*BLOSUM*62 _and *S*_*profile *_with 41 windowed residues. From the figure we note that if we combined *S*_*BLOSUM*62 _and *S*_*profile*_, the performance with *S*_*profile *_(41 windowed residues) was increased more than other two cases even though it showed worst performance when we considered *S*_*profile *_score alone. *S*_*combined *_with *S*_*profile *_(41 windowed residues) detected 1613 true matches up to 1000 false matches, while considering 19 and 7 windowed residues recognized 1583 and 1485 true matches, respectively. The results indicate that considering both short and long-range properties important to increase search sensitivity. When we search phosphorylation peptides the most important property may be physicochemical properties of adjacent residues to a phosphorylation site. However, together with this information, considering long-range region similarities can provide evolutionary or structural similarities surrounding kinase binding sites. Thereby both properties effectively contribute to measures peptide similarities.

### Performance comparison with AutoMotif

Our new method was applied to 48 kinase groups, and the performance of 36 groups was compared to the benchmarked results of AutoMotif. The performance data of AutoMotif were extracted from AutoMotif web server. The mean accuracy, precision, and recall of AutoMotif with a polynomial kernel were 0.91, 0.47, and 0.17, respectively, while those of the new method were 0.93, 0.67, and 0.40, respectively. The performance variations among 48 kinase groups are shown in Table 3. We note that in general the new method shows better performance than AutoMotif. Especially, 22 kinase groups showed overall increased performance from AutoMotif in all three aspects (accuracy, precision, and recall).

**Table 3:** Performance comparison with AutoMotif for 48 kinases.

Kinase	New method			AutoMotif			Kinase	New method			AutoMotif		
	ACC	P	R	ACC	P	R		ACC	P	R	ACC	P	R
CDK_group(102)	0.93	0.57	0.85	0.94	0.80	0.46	**Abl(48)**	**0.92**	**0.67**	**0.25**	**0.89**	**0.00**	**0.00**
GSK-3_group(33)	0.91	0.56	0.15	0.91	1.00	0.03	**PDK-1(35)**	**0.96**	**0.85**	**0.66**	**0.93**	**0.82**	**0.32**
**PLK1(42)**	**0.90**	**0.20**	**0.02**	**0.90**	**0.00**	**0.00**	**PKA alpha(31)**	**0.94**	**0.92**	**0.39**	**0.92**	**0.83**	**0.15**
**GRK_group(37)**	**0.91**	**0.67**	**0.05**	**0.89**	**0.00**	**0.00**	IKK_group(35)	0.89	0.27	0.11	0.91	0.00	0.00
**EGFR(61)**	**0.92**	**0.66**	**0.34**	**0.88**	**0.00**	**0.00**	**CaM-KIIalpha(34)**	**0.92**	**0.63**	**0.18**	**0.90**	**0.00**	**0.00**
**MAPK14(50)**	**0.92**	**0.57**	**0.50**	**0.90**	**0.22**	**0.04**	**GSK-3beta(52)**	**0.91**	**0.63**	**0.10**	**0.90**	**0.20**	**0.02**
**InsR(45)**	**0.92**	**0.60**	**0.36**	**0.83**	**0.60**	**0.07**	CaM-KII_group(55)	0.92	0.63	0.18	0.92	0.89	0.15
CK2_group(248)	0.94	0.75	0.56	0.94	0.83	0.40	PKB_group(84)	0.94	0.87	0.46	0.95	0.87	0.57
AMPK_group(38)	0.93	0.86	0.32	0.91	1.00	0.06	CDK1(147)	0.94	0.63	0.84	0.92	0.65	0.28
**MAPKAPK2(34)**	**0.92**	**0.62**	**0.24**	**0.90**	**0.00**	**0.00**	**CDK2(78)**	**0.93**	**0.61**	**0.78**	**0.91**	**0.45**	**0.07**
**CK1_group(33)**	**0.91**	**0.50**	**0.12**	**0.91**	**0.00**	**0.00**	**CK2 alpha(127)**	**0.94**	**0.77**	**0.52**	**0.93**	**0.73**	**0.32**
PKA_group(330)	0.95	0.83	0.56	0.96	0.90	0.58	**Lyn(48)**	**0.93**	**0.81**	**0.27**	**0.88**	**0.00**	**0.00**
PKC alpha(188)	0.93	0.75	0.27	0.92	0.78	0.11	RSK_group(23)	0.95	0.81	0.57	No data		
**Syk(51)**	**0.94**	**0.75**	**0.53**	**0.86**	**0.30**	**0.07**	DNA-PK(21)	0.93	0.60	0.57			
**Fyn(49)**	**0.91**	**0.42**	**0.10**	**0.90**	**0.00**	**0.00**	Aurora(55)	0.93	0.81	0.31			
MAPK_group(52)	0.93	0.62	0.50	0.93	1.00	0.22	Met(26)	0.95	0.69	0.85			
MAPK3(88)	0.94	0.68	0.69	0.95	0.88	0.55	PHK_group(21)	0.92	0.67	0.29			
**MAPK1(117)**	**0.95**	**0.67**	**0.76**	**0.93**	**0.67**	**0.42**	GRK-2(29)	0.92	1.00	0.10			
**MAPK8(36)**	**0.93**	**0.59**	**0.61**	**0.90**	**0.00**	**0.00**	ROCK_group(23)	0.92	0.67	0.26			
**Lck(54)**	**0.93**	**0.94**	**0.30**	**0.90**	**0.00**	**0.00**	FGFR1(23)	0.90	0.42	0.22			
PKC_group(236)	0.93	0.76	0.26	0.93	0.85	0.24	PDGFR(21)	0.94	0.83	0.48			
**Src(154)**	**0.92**	**0.61**	**0.24**	**0.90**	**0.05**	**0.01**	CK1(39)	0.92	0.62	0.21			
**IGF1R(31)**	**0.92**	**0.69**	**0.58**	**0.84**	**0.50**	**0.09**	CDK5(22)	0.95	0.76	0.59			
ATM(57)	0.95	0.85	0.60	0.97	0.91	0.75	PAK1(28)	0.91	0.60	0.11			

ACC, P, and R indicate accuracy, precision, and recall, respectively. The mean accuracy, precision, and recall of the new method for 36 kinases are 0.93, 0.67, and 0.40, respectively while those of AutoMotif are 0.91, 0.47, and 0.17, respectively. The values in brackets represent number of known phosphorylation sites in the reference set. Kinase groups which show better performance in our method compared to AutoMotif are bolded.

From the individual performance variations, it is notable that the new method was more effective for the kinase groups that contain relatively a small number of positive peptides. For the large kinase groups that contain more than 100 positive peptides, such as CDK-group, CK2-group, PKA-group, PKC-alpha, MAPK1, PKC_group, Src, and CK2-alpha, only in three kinase groups (MAPK1, Src, and CK2-alpha) our new method showed the performance enhancement in all three aspects, while in a majority of small kinase groups that contain less than 100 positive peptides, the new method achieved significantly better performance than AutoMotif. This tendency of performance improvement depending on the number of positive peptides may have stemmed from the amount of information. If we have many positive peptides, we can easily recognize phosphorylation peptides since the search space of phosphorylation peptides is dense. On the other hand, if we do not have enough number of positive peptides, prediction methods based on the sequence information of positive peptides inevitably contain much noise, making the predictions unreliable. In this situation applying our new method effectively reduces noise and retrieves weak signals, resulting in high search sensitivity. In this regard we expect that we can apply the new method to other small PTM groups and equally achieve performance enhancement.

Moreover, we conducted 10-fold cross validation to measure the performance variation. The results are shown in Table 4. The mean accuracy, precision, and recall (0.93, 0.59 and 0.43, respectively) were comparable or slightly degraded from LOOCV of the new method, but still the performance was better than AutoMotif (LOOCV). The result indicates that the new method performs well in an independent query set.

**Table 4:** Average performance of 10-fold cross validation.

Kinase	New method			Kinase	New method			Kinase	New method		
	ACC	P	R		ACC	P	R		ACC	P	R
CDK_group	0.92	0.60	0.88	MAPK3	0.93	0.61	0.73	CDK1	0.94	0.63	0.90
GSK-3_group	0.88	0.32	0.17	MAPK1	0.94	0.66	0.80	CDK2	0.94	0.65	0.87
PLK1	0.90	0.00	0.00	MAPK8	0.91	0.53	0.83	CK2 alpha	0.95	0.76	0.66
GRK_group	0.91	0.15	0.07	Lck	0.92	0.75	0.20	Lyn	0.92	0.67	0.23
EGFR	0.92	0.69	0.42	PKC_group	0.92	0.76	0.30	RSK_group	0.96	0.80	0.85
MAPK14	0.93	0.67	0.60	Src	0.92	0.69	0.27	DNA-PK	0.88	0.40	0.35
InsR	0.93	0.62	0.45	IGF1R	0.95	0.76	0.70	Aurora	0.92	0.58	0.26
CK2_group	0.94	0.71	0.58	ATM	0.96	0.87	0.70	Met	0.96	0.80	0.85
AMPK_group	0.94	0.60	0.27	Abl	0.93	0.77	0.30	PHK_group	0.90	0.10	0.05
MAPKAPK2	0.92	0.63	0.33	PDK-1	0.96	0.86	0.67	GRK-2	0.94	0.30	0.15
CK1_group	0.90	0.20	0.10	PKA alpha	0.95	0.78	0.57	ROCK_group	0.94	0.70	0.35
PKA_group	0.95	0.81	0.59	IKK_group	0.91	0.43	0.20	FGFR1	0.89	0.10	0.05
PKC alpha	0.92	0.69	0.28	CaM-KIIalpha	0.94	0.65	0.37	PDGFR	0.94	0.70	0.45
Syk	0.94	0.86	0.54	GSK-3beta	0.90	0.32	0.12	CK1	0.93	0.55	0.23
Fyn	0.90	0.25	0.13	CaM-KII_group	0.92	0.62	0.20	CDK5	0.97	0.87	0.75
MAPK_group	0.92	0.64	0.54	PKB_group	0.94	0.93	0.41	PAK1	0.94	0.50	0.25

The mean accuracy, precision, and recall of the new method for 48 kinases are 0.93, 0.59, and 0.43, respectively.

### Performance assessment with independent test set

We also benchmarked our results to six existing methods, GPS [14], KinasePhos [10], NetPhosK [6], PPSP[11], PredPhospho [7], and Scansite [22] by using independent test set created by Wan et al. [19]. The test set consists of four main kinase families (CDK, CK2, PKA, and PKC) and contains phosphorylation sites derived from Phospho.ELM, PhosphoSite, and Swiss-Prot. The advantage of new test set is unbiased and independent data, and thereby we can fairly compare several different algorithms. To assess performance of new test set we generated new reference set by using remaining data after removing new test set from PhosPho.ELM.1208. We evaluated the performance by comparing area of under ROC curves. The results of the six existing methods reported by Wan et al. [19] were used.

The area of under ROC curves are shown in Table 5. In table, top two ranked methods are bolded. We noted that our method was ranked as top or second in four kinases families. Our method showed better performance than GPS, KinasePhos, PredPhospho, and Scansite. Especially, compared to GPS designed to search phosphorylation peptides through sequence similarity using BLOSUM62 matrix, the performance improvement in our method indicates that addressing evolutionary information could be helpful to identity phosphorylation peptides. Furthermore, compared to PPSP our method shows significant improved results in CK2 family (6% increased) but similar performance in other kinase families. To conclude it is hard to say new method is outstanding compared to other methods but our method is generally effective to recognize phosphorylation peptides in four main kinase families.

**Table 5:** Performance variation with seven methods. The scores indicate the area under the ROC curves

	CDK	CK2	PKA	PKC
Noise-reducing	**0.8826**	**0.9288**	**0.8817**	**0.7995**
GPS	**0.8761**	0.8130	0.8446	0.7574
KinasePhos	0.8713	0.7508	0.8234	0.7440
NetPhoK	0.7767	**0.9307**	0.8749	0.7581
PPSP	0.8721	0.8767	**0.8860**	**0.7994**
PredPhospho	0.8670	0.7791	0.8537	0.7149
Scansite	0.7584	0.7734	0.7656	0.6397

### Web server construction

**Figure 3:**
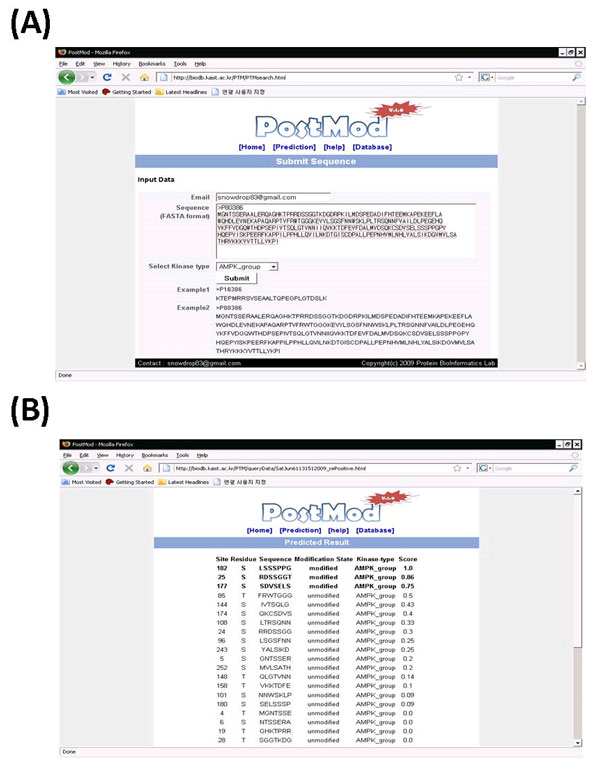
**The PostMod server input page (A) and result page (B)**. In input page, search sequence is pasted into the text box and one of 48 kinase types is selected. The default kinase type is AMPK_group. The example sequence is AMPK beta-1 chain (UniProt id is P80386). The search result of phosphorylation sites are shown in (B). There are 36 candidate phosphorylation sites (S, T) and three of them are recognized as phosphorylation sites (bolded line).

We constructed the web server to provide an easy access to our new phosphorylation site prediction method. Our web server, PostMod (prediction of Post-translational Modification sites) was implemented with python CGI scripts and html. Currently we provide prediction of phosphorylation sites for 48 kinases but our future direction is to apply the new method to other kinds of PTM sites. Figure 3 shows the sever input (A) and output pages (B). The search sequence can be submitted by pasting it into the text box. The server allows a user to select one of 48 different kinase types. For the query sequence the server searches all putative phosphorylation sites and generates several peptides. Meanwhile, the server generates PSSM and PSFM matrix of the query sequence by executing blastpgp 2.2.20. After that, the sever compares sequence similarity with the peptide sequences in the reference set which are phosphorylation and non-phosphorylation sites of the corresponding kinase type from phospho.ELM database. Figure 3(B) is the output page for AMPK beta-1 chain (UniProt id is P80386). All putative 36 phosphorylation sites (S, T) were shown together with confidence scores. Three predicted phosphorylation sites were bolded. The confidence score is the fraction of phosphorylation peptides among the top β hits (see Method section). We set the threshold value for phosphorylation sites as 0.5 of confidence score. User can receive search result via email. The web server is available through our website [23].

## Conclusion

The present method is remarkable in the sense that it is simple and computationally costless, and yet shows the outstanding performance improvement for the various kinds of kinases. We showed that when we combined the BLOSUM62 matrix-based similarity measure and the profile-profile alignment scores, the recognition results were significantly improved. Moreover, applying our noise-reducing system by exploiting indirect relationships effectively eliminated noise, and thereby increased the overall performance. The overall performance enhancement on 48 kinds of different kinases suggests that our method is generally applicable to other types of PTMs. Furthermore, it is expected that combining our method with better similarity methods would achieve higher accuracy for finding phosphorylation sites.

Performance degradation in a conventional sequence similarity measures is mainly originated from improper similarity scoring system, which gives higher scores to unrelated peptides, producing many false positives. The best solution may be developing new features which well discriminate positives from negatives. If we do not have such powerful features, we need to concentrate on removing noise. In this manner we addressed a concept of indirect relationships, and we showed that considering indirect relationships can be a powerful tool to eliminate the false positives.

To conclude, applying the new method produces good results without need of sophisticated machine learning techniques in detecting phosphorylation sites. Furthermore, we expect that applying our new method to other kinds of biological analysis would achieve high performance improvement.

## Competing interests

The authors declare that they have no competing interests.

## Authors' contributions

I.J, A.M, M.Y, and D.K designed the methods, and experimental setup. I.J carried out the implementation of the all methods. I.J wrote the manuscript under D.K, A.M and M.Y's technical supervision and mentorship. All authors have read and approved the final manuscript.
